# The protective effect of Buffalo’s milk against toluene induced-nephrotoxicity in rats

**DOI:** 10.15171/jnp.2017.30

**Published:** 2016-12-17

**Authors:** Maryam Afravy, Kambiz Angali, Ali Khodadadi, Massumeh Ahmadizadeh

**Affiliations:** ^1^Department of Occupational Health, Engineering, Ahvaz Jundishapur University of Medical Sciences, Ahvaz, Iran; ^2^Department of Statistics and Epidemiology, School of Health, Ahvaz Jundishapur University of Medical Sciences, Ahvaz, Iran; ^3^Department of Immunology, Faculty of Medicine, Ahvaz Jundishapur University of Medical Sciences, Ahvaz, Iran; ^4^Physiology Research Centers, Ahvaz Jundishapur University of Medical Sciences, Ahvaz, Iran

**Keywords:** Antioxidant enzymes, Buffalo’s milk, Toluene, Kidney, Rat

## Abstract

**Background:**

Toluene is widely used in different activities of industrial, commercial and household applications. It can cause damage to the human body. Buffalos’ milk has a good nutritive value.

**Objectives:**

The aim of this study is to examine the negative effects of toluene on kidney tissues and to investigate the protective effects of buffalo’s milk against toluene-induced nephrotoxicity in rats.

**Materials and Methods:**

Forty adult male Wistar rats (180-220 g weight) were randomly assigned to eight groups (n = 5). Animals in groups I to IV received oral gavage 1 mL distilled water (DH_2_O) and groups V to VIII received oral gavage 1 mL buffalo’s milk. Ten minutes later, animals were received toluene (i.p) at doses of 300 mg/kg (groups I and V), 600 mg/kg (groups of II and VI), and 900 mg/kg (groups of III and VII), respectively. The animals in groups IV (control) and VIII were injected vehicle (corn oil) only. The experiment repeated for seven consecutive days. Twenty-four hours after the last administration, animals were killed with overdose of sodium pentobarbital. Blood samples were analyzed for blood urea nitrogen (BUN) and creatinine (Cr). One part of the kidney tissues were excised for measuring the activities of superoxide dismutase (SOD), glutathione reductase (GR) and catalase (CAT) and the level of malondialdehyde (MDA). Another parts were excised for histopatholgical examination.

**Results:**

Administration of toluene to male rats produced dose-dependent damage in the kidney. This was noted by elevation of BUN, Cr and MDA levels. In contrast, diminished the CAT, GR and SOD enzyme activities in rats treated with toluene when compared to those in control animals. Histopathological manifestations were also observed in dose related manner in toluene-treated rats. Buffalo’s milk had no effect on the biochemical parameters and kidney morphology when compared to those in control. However, it was able to prevent rat kidney against toluene toxicity.

**Conclusions:**

The results of this study demonstrated that toluene damages kidney tissue and is a nephrotoxic substance. Buffalo’s milk was able to prevent the renal damage as an antioxidant and a nephroprotective agent.

**Implication for health policy/practice/research/medical education:**

In an experimental study, we found that buffalo’s milk as an antioxidant agent protects kidney against toluene induced nephrotoxicity. The mechanism of these renoprotective effects mainly includes amelioration of lipid peroxidation produced by toluene as well as elevation antioxidants enzymes activities by this milk.

## 1. Background

Toluene with C_7_H_8_ formula is an aromatic hydrocarbon. It is largely used all over the world as volatile organic compounds and produced in large quantities to be used in different activities of industrial and commercial applications including paints, varnishes, inks, adhesives, plastics (1,2). Toluene is usually an abused organic solvent and is highly potential for different kinds of abuse (3). Exposure to toluene may cause damages to variety organs including liver, kidney, lung, heart and nervous system (4).

Toluene is metabolized to hippuric acid in laboratory animals. Studies on industrially exposed workers to toluene have shown similar patterns of metabolism of this chemical as to those observed in experimental animals (5). Studies in both humans and animals have shown that the majority of toluene in the body is eliminated in the urine, mainly as metabolites (6,7). It has been recognized that oxidative stress plays important and significant role in toluene produced renal injury (8). Kidney is the main organ for toluene excretion. Nephrotoxicity is a concern over occupational hazards associated with toluene has led to considerable interest to study of this agent on experimental animals.

The association between food and health is well established. Recently, much attention has focused on naturally occurring antioxidant for preventing of xenobiotics induced oxidative damage in living system. Meydan et al showed that toluene hurts kidney tissue and this could be prevented by caffeic acid phenethyl ester (8). It has been shown bovine milk has antioxidant capacity, represented by the natural occurring of vitamin E and C, beta-carotene and enzymatic systems (9-11). Buffalo’s milk has high concentrations of proteins, fat, lactose, minerals and vitamins (12). We found that buffalo’s milk protected rats against xylene-induced hepatotoxicity and nephrotoxicity (13). However, to our knowledge the effect of buffalo’s milk against adverse effects of toluene has not been reported previously.

## 2. Objectives

The aim of the present study is to determine the effects of buffalos’ milk on toluene-induced nephrotoxicity.

## Materials and Methods

### 3.1. Chemicals

All reagents and chemicals were of analytical grade or higher purity. Toluene was purchased from Aldrich-Chemical Co., thiobarbituric acid (TBA), sodium pentobarbital obtained from Sigma Chemical Co. 1,1,3,3 tetraethoxypropane (TEP) purchased from Merck Chemical Co.

### 3.2. Animal treatments

Adult male Wistar rats (250-300 g) were housed in groups of three in clear polypropylene cages in a light cycle (12-hour light and 12-hour dark) and temperature-controlled room. The animals were allowed food and tap water ad libitum.

### 3.3. Buffalo’s milk

Buffalo’s milk samples were collected daily early in the morning from the herd of buffalos by hand milking as normally practiced by the farmers. The samples were collected in sterile screw bottle and kept in a cool box until being transported. The rats were given this fresh milk by oral delivery (gavage) 1 mL/animal as such without any further treatment.

### 3.4. Study design and treatments

Forty adult male Wistar rats (180-220 g weight) were randomly assigned to eight groups (n = 5). Animals in groups I to IV received oral gavage 1mL distilled water (DH_2_O) and groups V to VIII received oral gavage 1 mL buffalo’s milk (BM). Ten minutes later, animals were received toluene (i.p) at doses of 300 mg/kg (groups I and V), 600 mg/kg (groups II and VI), and 900 mg/kg (groups of III and VII), respectively. The animals in groups IV (control) and VIII were given vehicle (corn oil) only. The experiment repeated for seven consecutive days. Twenty-four hours after last administration, animals were killed with overdose of sodium pentobarbital. Blood was collected for determination of blood urea nitrogen (BUN) and serum creatinine (Cr) levels. Kidney tissues were removed washed with normal saline and excised for estimation of enzymatic activities of superoxide dismutase (SOD), glutathione reductase (GR), catalase (CAT) and malondialdehyde (MDA) levels. Other parts of the kidney tissues were excised for histopathological examination.

### 3.5. Biochemical studies

#### 3.5.1. Renal function tests

BUN and serum Cr were determined by diacetyl monoxime and Jaffe methods respectively (14,15).

#### 3.5.2. Antioxidant enzymes activities

Kidney tissues were collected for determination of SOD, CAT and GR activities. Theses antioxidant enzymes were assessed using a commercially available kits (Biovision) according to the manufacturer’s instructions.

#### 3.5.3. Peroxidation markers

Malondialdeyde (MDA), the product of lipid peroxidation, was estimated by the method described by Buege and Aust (16). Tissues lipid peroxidation was measured in whole-kidney homogenate at 10 000 g for 10 minutes, the supernatant was taken. Aliquots (1 mL) were analyzed for MDA content after the addition of 2 mL of TBA reagent. Then tube was in vortex mix for 10 seconds and placed in a boiling water bath (90-100°C) for 20 minutes. After cooling for 7 minutes, the resulting supernatant was removed and measured at wave of 532 nm with the use of the SERIEC-7000 spectrophotometer. MDA concentration was determined by using 1,1, 3, 3-tetraethoxypropane as external standard (0.5-2.5 μM).

### 3.6 Histopathological studies

The part of kidney tissues was removed, fixed and processed for light microscopy, using hematoxylin-eosin (H&E) staining technique. Five histological sections each at least 15 μm apart were taken from each tissue block and stained with H&E. The criteria for cell injury included nuclear dilation, loss of staining capacity and obvious cellular swelling.

### 3.7. Ethical issues

The research was approved by ethical committee of Jundishapur University of Medical Sciences. Prior to the experiment, the protocols were confirmed to be in accor­dance with the guidelines of Animal Ethics Committee of Jundishapur University of Medical Sciences.

### 3.8. Statistical analysis

Biochemical data were expressed as mean ± standard error (SE) of five animals. The results were analyzed by analysis of variance (ANOVA); completely randomized design, and treatment differences were identified by the method of Newman-Keuls and *P* value ≤ 0.05 was determined as the criterion for significance.

## 4. Results

**Figure 1:**
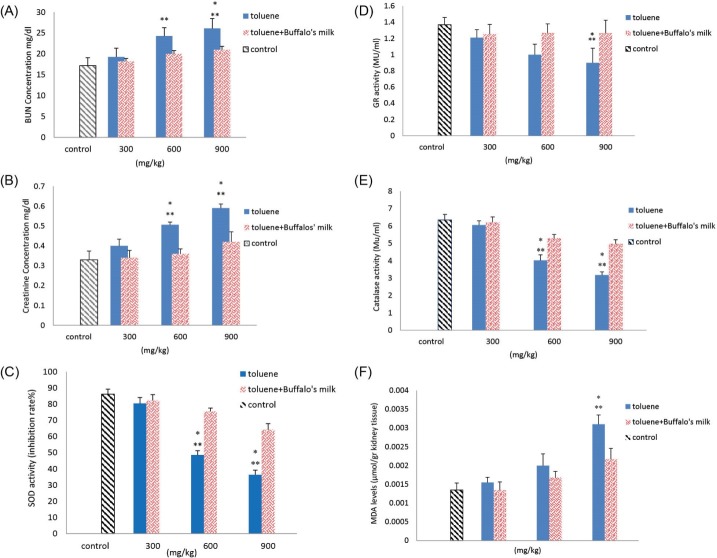
Effects of buffalo’s milk on (A) BUN levels, (B) Cr levels, (C) SOD activity, (D) GR activity, (E) catalase activity, and (F) MDA levels in rat kidney tissues treated with toluene. *Significantly different from rats pretreated with buffalo’s milk and were given the same dose of toluene (*P* ≤ 0.05). **Significantly different from control (*P* ≤ 0.05). Abbreviations: BUN, blood urea nitrogen; Cr, creatinine; GR, glutathione reductase; MDA, malondialdehyde; SOD, superoxide dismutase.

A dose related increase in BUN level was noted in toluene treated rats. The level of BUN significantly increased in animals administrated toluene at doses of 600 and 900 mg/kg when compared to control rats. Buffalo’s milk had no effect on BUN when compared to control animals. However, the level of BUN significantly decreased in animals pretreated with buffalo’s milk and received 900 mg/kg toluene when compared to non-pretreated rats and exposed the same dose of toluene (Figure 1A). Similarly, dose-dependent elevation of Cr was noted in toluene treated rats when compared with rats in the control group. Elevation of Cr was significant in rats treated with toluene at doses of 600 and 900 mg/kg when compared to those in control rats. Pretreated rats with buffalo’s milk significantly decreased Cr levels in animals exposed to 600 and 900 mg/kg toluene when compared non-pretreatment rats received the same dose of toluene (Figure 1B).

Reduction of SOD and CAT enzyme activities was found to be statistically significant in rats following treated with 600 mg/kg and 900 mg/kg toluene and GR enzyme activity in rats treated with 900 mg/kg toluene when compared to those in control animals (Figure 1C- 1E). Pretreated rats with buffalo’s milk significantly increased the activities of these enzymes when compared to non-pretreatment rats and received the same dose of toluene (Figure 1C- 1E).

Kidney MDA levels increased in rats treated with various doses of toluene and it was significant in animals treated with 900 mg/kg. Pretreated rats with buffalo’s milk significantly decreased MDA levels when compared non-pretreatment rats and received the same dose of toluene (Figure 1F).

**Figure 2:**
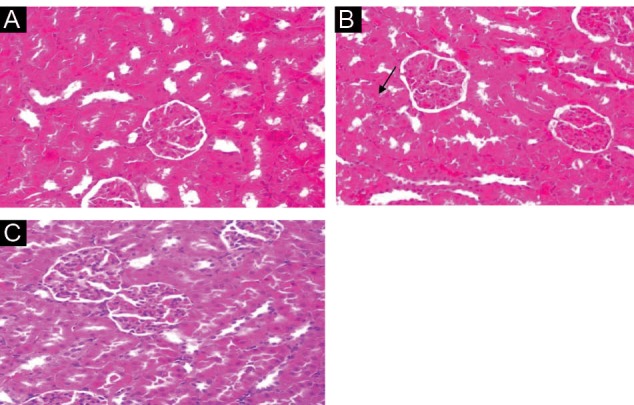
(A) Light micrograph of rat kidney treated with vehicle (control) showing the normal architecture of renal tissue (H&E ×200). (B) Light micrograph of rat kidney treated with 900 mg/kg toluene, showing renal tubular cells were swollen, had loss of staining capacity. Disorganization and congestion are apparent (arrow) (H&E ×200). (C) Light microscopy of kidney of rats pretreated with buffalos’ milk and subjected to 900 mg/kg toluene. Photo, showing toluene-induced nephrotoxicity markedly diminished in comparison with non-pretreated animals which received the same dose of this chemical (Figure 2B) (H&E ×200).

Administration of vehicle alone did not produce detectable injury in rat kidney (Figure 2A). However, dose-related injury in toluene-treated rats were noted. Light microscopy revealed that renal tubular cells were swollen, had loss of staining capacity, and nuclei appeared to be dilated, presence of blood clot was noted in toluene-treated rats (Figure 2B). Similarly, buffalo’s milk had no effect on kidney cells. However, the extant of toluene-induced injury decreased in buffalo’s milk pretreated rats when compared to those treated with toluene only (Figure 2C).

## 5. Discussion

The study of the effect of toluene on experimental animals may be useful for better understanding of the clinical pictures following toluene exposure in humans. Our data showed that, toluene induced dose dependent damage in rat kidney. Assessment of renal injury was estimated by determination of BUN and Cr levels and histopathological examination. Elevation of biochemical parameters (BUN and Cr) and histopathological damage in the kidney were reported in rats exposed to toluene (8,17). Occupational exposure to toluene was reported to be associated with development and progression of renal failure (18-20). Toluene inhalation is associated with various severe metabolic alterations, including renal tubular acidosis, hypokalemic paralysis and profound metabolic acidosis (21). Ketan et al reported significant decline in the activities of SOD and CAT with concomitant increase in lipid peroxidation in mice exposed to benzene, toluene, xylene (22). Taverner et al reported acute renal failure due to interstitial nephritis induced by toluene (19). Meydan et al showed intraperitoneal injection of toluene in rats with a dose of 500 mg/kg caused alterations of SOD and CAT as well as induced histopathological damage in rat kidney (8). We observed significant increase MDA concentration and decreased antioxidant enzymes (CAT, GR, SOD) activities in kidney tissues of rats following exposure to various doses of toluene when compared to those in control animals.

Our findings along with other reports further support the view that toxic effects of toluene appears to be mediated at least in part by free radical generation.

The effects of milk on human health have been studied for years. However, the relationship between dairy products and xenbiotics induced adverse effects still inconclusive. Chuang et al suggested milk protected workers against lead induced neuropathy (23). Gomes et al demonstrated intake milk and dairy products modulated level of lead in blood in workers highly exposed to the metal (24).

Recent studies showed that camel’s milk has potential to prevent kidney against chemical-induced injury. Al-hashem reported that camel’s milk protects rat kidney againt aluminum chloride-induced toxicity (25). Similarly, Afifi observed pretreated of mice with camel’s milk diminished cisplatin-induced nephrotoxicity (26). Our biochemical and histopathlogical study indicated buffalo’s milk caused a recovery of kidney damage induced by toluene. This was noted by remarkable restoration of Cr, BUN, MDA and increased activities of antioxidant enzymes (SOD, GR, CAT) in toluene trated rats. The productive effect of buffalo’s milk against toluene–induced oxidative stress and kidney tissue damage at least in part could be attributed to its antioxidant property. Keeping in mind that toluene is an organic solvent with extensive industrial applications and the considerable potential of occupational exposure, this study may lead to a better understanding of the influence of milk on toluene induced-nephrotoxicity.

## Conclusions

Our results showed that toluene is capable of producing marked alterations in biochemical parameters, histopathological changes and oxidative damage, and diminishing activity of kidney antioxidant enzymes. Administration of buffalo’s milk prior exposure rat to toluene protected kidney against toluene-induced nephrotoxicity. The protective effect of buffalo’s milk could be attributed to its antioxidant activity.

## Conflicts of interest

The authors declared no competing interests.

## Authors’ contribution

MA provided technical assistance, collection and preparation of the manuscript. KA analyzed the data. AK supervised antioxidant’s biochemical data. MA designed, supervised the study and prepared the final draft of the article .

## Funding/Support

This study was supported by Physiology Research Center and the research deputy of Ahvaz Jundishapur University of Medical Sciences (Grant #APRC-93-07).

## Acknowledgments

The authors wish to thank the research deputy of Ah­vaz Jundishapur University of Medical Sciences for offering the grants for this investigation. The source of data used in this paper was from master thesis of Maryam Afravy, student of Occupational Health Engineering Department, School of Health, Ahvaz Jundishapur University of Medical Sciences, Ahvaz, Iran. Our special thanks go to Dr. B. Mohammadian for reviewing histopathological samples.
